# Participation of the spleen in the IgA immune response in the gut

**DOI:** 10.1371/journal.pone.0205247

**Published:** 2018-10-04

**Authors:** Desiree Weiberg, Marijana Basic, Margarethe Smoczek, Ulrike Bode, Melanie Bornemann, Manuela Buettner

**Affiliations:** 1 Institute of Functional and Applied Anatomy, Hannover Medical School, Hannover, Germany; 2 Department of Nuclear Medicine, Hannover Medical School, Hannover, Germany; 3 Institute for Laboratory Animal Science, Hannover Medical School, Hannover, Germany; Ohio State University, UNITED STATES

## Abstract

The role of the spleen in the induction of an immune response to orally administered antigens is still under discussion. Although it is well known that after oral antigen administration specific germinal centres are not only formed in the Peyers patches (PP) and the mesenteric lymph nodes (mLN) but also in the spleen, there is still a lack of functional data showing a direct involvement of splenic B cells in an IgA immune response in the gut. In addition, after removal of mLN a high level of IgA+ B cells was observed in the gut. Therefore, in this study we analysed the role of the spleen in the induction of IgA+ B cells in the gut after mice were orally challenged with antigens. Here we have shown that antigen specific splenic IgM+ B cells after *in vitro* antigen stimulation as well as oral immunisation of donor mice were able to migrate into the gut of recipient mice, where they predominantly switch to IgA+ plasma cells. Furthermore, stimulation of recipient mice by orally administered antigens enhanced the migration of the splenic B cells into the gut as well as their switch to IgA+ plasma cells. Removal of the mLN led to a higher activation level of the splenic B cells. Altogether, our results imply that splenic IgM+ B cells migrate in the intestinal lamina propria, where they differentiate into IgA+ plasma cells and subsequently proliferate. In conclusion, we demonstrated that the spleen plays a major role in the gut immune response serving as a reservoir of immune cells that migrate to the site of antigen entrance.

## Introduction

In the gut, the mucosal immune system can be divided into inductive and effector sites [1]. Mucosal inductive sites include the gut-associated lymphoid tissue (GALT), for instance the Peyer´s patches (PPs), and the mesenteric lymph nodes (mLN) [1], whose characteristic feature is to initiate a preferential adaptive immune response in the form of immunglobulin A (IgA) production [2]. To initiate the adaptive immune response, after penetrating the intestinal mucosa pathogens are encountered by dendritic cells (DCs) and then transported to the mLN [3]. However, particular antigens may be first detected in the Peyer´s patches (PPs) and subsequently transferred to mLN [1]. PPs and mLN belong to the secondary lymphoid tissues in which the immune response is initiated [4]. In these sites DCs present mucosa sampled antigens (Ags) to T cells leading to their activation followed by a clonal expansion [5]. Upon clonal expansion majority of effector T cells leave the T cell area, enter the circulation and settle in the periphery, where they contribute to the coordination of the immune response. However, some of these cells migrate into the B cell area to support the activation of B cells. Activated B cells leave mLN by entering the blood stream and lymph, migrate into mucosal effector sites such as intestinal lamina propria and differentiate into plasma cells, which secrete predominantly IgA [2].

The spleen is the largest secondary lymphoid organ directly connected to the blood stream. It consist from the red pulp, which filters the blood for senescent erythrocytes, and the white pulp, which detects blood-borne Ags and protects against systemic infection [6]. The importance of the spleen in the defence against certain bacteria such as pneumococci or meningococci was recognized in the splenectomised patients [7]. Ags enter the spleen either as soluble Ags or are presented by macrophages [8] or DCs, which migrate into the spleen [9, 10]. In the periarteriolar sheath (PALS) T cells are activated by recognizing the presented Ags. Splenic effector T cells, similarly as in lymph nodes, migrate into the circulation or into B cell follicles, where they support B cell activation [9]. Specialized marginal-zone macrophages and marginal-zone B cells represent the first line of defence against viruses and encapsulated bacteria [9, 11, 12]. Marginal-zone B cells express high levels of IgM and CD21, but do not express IgD. This cell surface marker expression pattern distinguishes them from follicular B cells that are characterized by intermediate expression of IgM, CD21 and IgD [13, 14]. Furthermore, marginal-zone B cells differentiate into plasma cells in response to T cell independent antigens and secrete low affinity IgM antibodies [15, 16]. In contrast, follicular B cells are mature B cells with a key role in the adaptive immune response, whose differentiation requires T cell support [13, 17]

Furthermore, many diseases have been associated with hyposplenism, a dysfunction of the spleen, such as gastrointestinal diseases, hepatic and autoimmune disorders [18]. This condition is characterized by Howell-Jolly bodies, monocytosis, lymphocytosis and increased numbers of platelets. One of the most well-known diseases associated with the development of hyposplenism is coeliac disease. Hyposplenism occurs in 25–75% coeliac disease patients and can increase the risk of sepsis in these patients [19–21].

To investigate the role of the spleen in the gut immune response our group established a model based on the removal of mLN in mice [22]. In this model pseudo-afferent lymphatic vessels develop after more than 10 days post mLN resection and transport cells and Ags from the draining area into the thoracic duct and blood [23, 24]. In our previous study we have demonstrated that in mLN resected mice cholera toxin (CT) oral challenge induced the immune response in the spleen characterized by B cell proliferation and formation of germinal centres [22]. Furthermore, CT-specific IgM antibodies were detected in the serum, whose presence strongly supported involvement of the spleen in the orally triggered immune response. Moreover, oral administration of CT increased the numbers of IgA+ B cells in the lamina propria of the small intestine [22]. In addition, several other studies have also shown that the spleen contributes to the mucosal immune response in the gut [21, 25–27].

Therefore, the aim of this study was to analyse whether splenic B cells are actively involved in the gut immune response. Furthermore, we examined whether the spleen responded to orally administered antigens and what was the role of mLN in this process. Finally, we determined the cell type participating in the antigen sampling and whether the gut mucosa sampled antigens, which induce the immune response in the spleen, are directly involved in the effective immune response.

## Materials and methods

### Animals

Female C57BL/6J and C57BL/6-Tg(CAG-EGFP)1Osb/J (eGFP) mice were bred at the Central Animal Facility of the Hannover Medical School. Mice were utilized for the experiments when their weight reached 18–25 g. C57BL/6-Ly.5.1 (Ly5.1) mice were obtained from Charles River (Belgium). The Ly system is a diallelic polymorphism of the CD45 molecular system [28]. This polymorphism can be monitored by an antibody against CD45.1 (Biolegend, Aachen, Germany). C57BL/6-Tg(IghelMD4)4Ccg/J were purchased from Jackson Laboratory. MD4 mice express a transgenic BCR for Hen Egg lysozyme (HEL). MD4 B cells can be identified using a specific antibody against the HEL specific IgM (IgM^a^).

### Ethical guidelines

This study was conducted in accordance with the German Animal Welfare Act and the European Directive, 2010/63/EU. All experiments were approved and permitted by the Lower Saxony State Office for Consumer Protection and Food Safety (LAVES, license: 09/1667 and 17/2564). Routine microbiological monitoring according to recommendations of the Federation of European Laboratory Animal Science Associations did not reveal any evidence of infection with common murine pathogens [29, 30]. Mice were maintained in a room with controlled environment: 20–24°C; relative humidity 55±5%; 14:10 h light:dark cycle and 12–14 air changes per hour. Pelleted diet (Altromin1324 TPF, Lage, Germany) and filtered distilled water were provided ad libitum. Mice were monitored for health and weight daily. Disinterest, diminished activity and reduced food intake were defined as a humane endpoint.

### MLN resection

MLN resection was performed under combined anaesthesia using Ketamine (Gräub AG, Bern, Switzerland) and Domitor (Pfizer, Karlsruhe, Germany). MLN were removed by micro-dissection along the length of the superior mesenteric artery to the aortic root as described previously [31, 32]. MLN resected mice developed pseudo-afferent lymphatics within 4 weeks post-surgery. In the control group, the intestine was taken out of the peritoneal cavity and returned after washing it with PBS (sham-surgery).

### Uptake of microspheres

Control and mLN resected group received 10^12^ green fluorescent microspheres (0.2 μm, Fluoresbrite YG, Polysciences Europe, Eppelheim, Germany) and 50μg CT (Sigma, Steinheim, Germany) by oral gavage. 4h later mLN and spleens were removed. Cell suspensions from these organs were stained with mAB CD11c PE (clone N418; 1:100; Miltenyi, Bergisch Gladbach, Germany), CD103 PerCP-Cy5.5 (clone 2E7; 1:100; Biolegend, San Diego, USA), F4/80 APC (clone BM8; 1:500; Biolegend) and CD19 APC-H7 (clone 1D3; 1:200; BD Biosciences, Heidelberg, Germany). The uptake of micropheres was analysed by flow cytometry as described previously [33].

### Isolation and cell transfer of splenic IgM+ B cells

Ly5.1 mice were gavaged with 250 μl ovalbumin (OVA, 100mg/ml; grade III; Sigma) and CT (40μg/ml, Sigma) as an adjuvant on day 0, 7, 14 and 21. Control group was left untreated. On day 25 mice were sacrificed by heart punction under isofluran anaesthesia (Baxter GmbH, Unterschleißheim, Germany) and spleens or mLN were removed, respectively (S1 Fig). Erythrocytes were lysed in the splenic cell suspension as described previously [34]. Splenic IgM+ B cells were isolated by positive selection using magnetic cell separation (anti-IgM beads, MACS, Miltenyi). To enhance the purity of IgM+ B cells the selection was repeated twice. Purified splenic IgM+ B cells were re-suspended with 150 μl PBS and intravenously (i.v.) injected into WT recipients (15 x 10^6^ cells/ mouse). Mice were divided in four groups (S1 Fig): Untreated groups (1. mLN intact and 2. mLN resected mice) and OVA treated groups (3. mLN intact and 4. mLN resected mice). In the OVA treated groups recipients received 100 mg OVA by oral gavage 1 day after the cell transfer. After 4 days all animals were sacrificed and the gut, PP and the spleen were collected and analysed by flow cytometry.

In addition, to evaluate the proliferation, bromodesoxyuridine (BrdU; 1.25mg BrdU/100 μl PBS; Sigma) was given in the drinking water between day 21–25 and i.v. on day 21 and 23.

For the adaptive transfer of splenic and mLN IgM+ B cells Ly5.1 and eGFP mice were treated as already described above. On day 25 mice were sacrificed, spleens or mLN were removed and IgM+ B cells were isolated. Identical cell numbers of splenic and mLN IgM+ B cells were injected into WT recipients. Furthermore, recipients received 100mg OVA or PBS (control group) by oral gavage 1 day after the cell transfer and were sacrificed 3 days later for flow cytometry analysis.

### *In vitro* stimulation

Splenic B cells from MD4 mice were enriched using MACS technique as described above. B cells were cultured for 24 hours in RPMI medium (Biochrom, Berlin, Germany) with 10% FCS (GE Healthcare Life Sciences, Buckinghamshire, UK), 1% Pen/Strep (Gibco, Thermo Fisher Scientific, Waltham, USA) and 0,3mg/ml Glutamin (Biochrom) containing 1μg HEL (Sigma) for stimulation. Subsequently, cells were i.v. injected into WT mice, which were simultaneously orally gavaged with 1mg HEL or PBS. One day later mice were sacrificed and analysed via flow cytometry.

### Preparation of a single-cell suspension from the small intestine

The small intestine was removed and rinsed with PBS. Subsequently, PPs were removed and the small intestine was cut open and sliced into thick section slices. These pieces were minced and then placed into a Hanks Salt solution (Biochrom) containing 5mM EDTA (Serva, Heidelberg, Germany), 1 mM DL-Dithiothreitol (Sigma) and 5% FCS for 20 minutes at 37C°. The suspension was subsequently filtered. This procedure was repeated three times. Cell suspensions were collected and pooled. Remaining slices were incubated in Hanks Salt solution containing 1.5 mg/ml collagenease VIII (Sigma) and 5% FCS. This cell suspension was filtered and added to the previously collected cell suspension. Cells were collected by centrifugation. After centrifugation cells were counted and analysed by flow cytometry.

### Preparation of a single-cell suspension from lymphoid organs

Cell suspensions were prepared from isolated PPs, mLNs and spleens. In addition, erythrocytes from the splenic cell suspension have been lysed as described previously [34]. Cells were collected after washing steps, counted and analysed by flow cytometry.

### Flow cytometry

B1 cells were detected via mAB IgM PerCP-Cy5.5 (clone R6-60.2; 1:100; BD Biosciences), CD19 APC-H7 (BD Biosciences), CD5-AlexaFlour647 (clone YTSS 121.5.2; 1:50; Serotec, Kidlington, UK) and CD11b (clone M1/70; 1:500; BD Biosciences), which was visualized by a secondary goat anti rat Ig polyclonal Ab (1:500; BD Biosciences) conjugated with PE. B2 cells were identified using IgM-PerCP-Cy5.5 and CD19 APC-H7. Furthermore, mAB CD21/35 APC (clone 7G6; 1:500; BD Biosciences), CD23 PE and IgD FITC (both kindly provided by R. Förster) were used for a more detail B cell characterisation. Around 1x10^6^ cells were incubated with mAB CD45.1/Ly.1 PE (clone A20; 1:100; Biolegend) or IgM^a^ FITC (clone DS-1; 1:500; BD Biosciences) to identify the injected cells. Subpopulations of B cells were identified by mAB B220 (clone RA3-6B2; 1:1000; BD Biosciences), IgM PerCP-Cy 5.5, IgD FITC, IgA (clone C10-3; 1:500; BD Biosciences), CD80 PerCy5.5 (clone 16-10A1; 1:1000; BD Biosciences) and CCR9 PE (clone eBIOCW-1.2; 1:100; eBioscience). IgA was visualized by a polyclonal secondary goat anti-rat Ig (1:500; BD Biosciences) conjugated with APC. Injected CD45.1/Ly.1 cells were analysed by counting more than 1000 positive cells/ organ. BrdU was detected as described previously [35]. Flow cytometric analyses were performed using the FACS Canto (BD Biosciences).

### Immunohistochemistry

Cytospins of isolated splenic IgM+ B cells and spleen cryosections were prepared as described previously [36]. Cytospins and cryosections were fixed in acetone solution (10 min, -20°C). The alkaline phosphatase anti-alkaline phosphatase (APAAP) technique was used to phenotype IgM+ (clone II/41; 1:10; BD Biosciences) B cells [37]. After incubation with the primary antibody slides were washed with TBS-Tween (0.05% Tween 20, Serva, Heidelberg, Germany) and incubated with the bridging antibody (rabbit anti-rat, 1:100 Dako, Hamburg, Germany) followed by the APAAP complex (Dako). As a substrate for the alkaline phosphatase Fast blue (Sigma) was used. All sections were counterstained with hemalaun and mounted in glycergel (Dako). BrdU^+^ cells were stained using peroxidase conjugated mAbs (clone BMG-6H8; 1:25; Roche Diagnostics, Mannheim, Germany) and visualized by diaminobenzidine substrate kit (Vector Laboratories, Burlingame, USA).

### ELISA

The serum was tested for specific Abs against OVA. For this, 0.5 mg OVA (grade VI, Sigma) was dissolved in 1 ml PBS. The plate was coated with 100 μl OVA/well over night at 4 C°. For washing TBS (Serva) containing Tween (TBS-Tween, Serva) was used. Wells were blocked with 5% milk powder (Heirler Cenovis GmbH, Radolfzell, Germany) dissolved in TBS-Tween for 1 h at 37 C°. Subsequently, wells were mounted with 100 μl of sample and incubated for 2h at 37 C°. OVA-specific IgA and IgM (all from BD Biosciences) were identified using biotin labelled antibody for 1h at room temperature. The specific Abs were visualized by incubation with horse-radish peroxidase coupled with streptavidine (BD Biosciences). As a substrate the Opt EIA substrate reagent A and B (BD Biosciences) were used. The reaction was stopped using the stopping solution (BD Biosciences). The optical density was measured in the ELISA-Reader Power Wave XS (Bio-Tek Instruments GmbH, Bad Friedrichshall, Germany) at the density of 405nm.

### Data analysis

Calculations, statistical analysis and graphs were done with the software Graphpad Prism 4.0 (Graphpad Software Inc., San Diego, USA). Mean and standard error of the mean were given in each graph. Statistical differences were calculated in the unpaired t-test and are indicated by *, P< 0.05; **, P< 0.01; ***, P< 0.001.

## Results

### Orally administered microspheres induce an increase of splenic B cells

**Fig 1:**
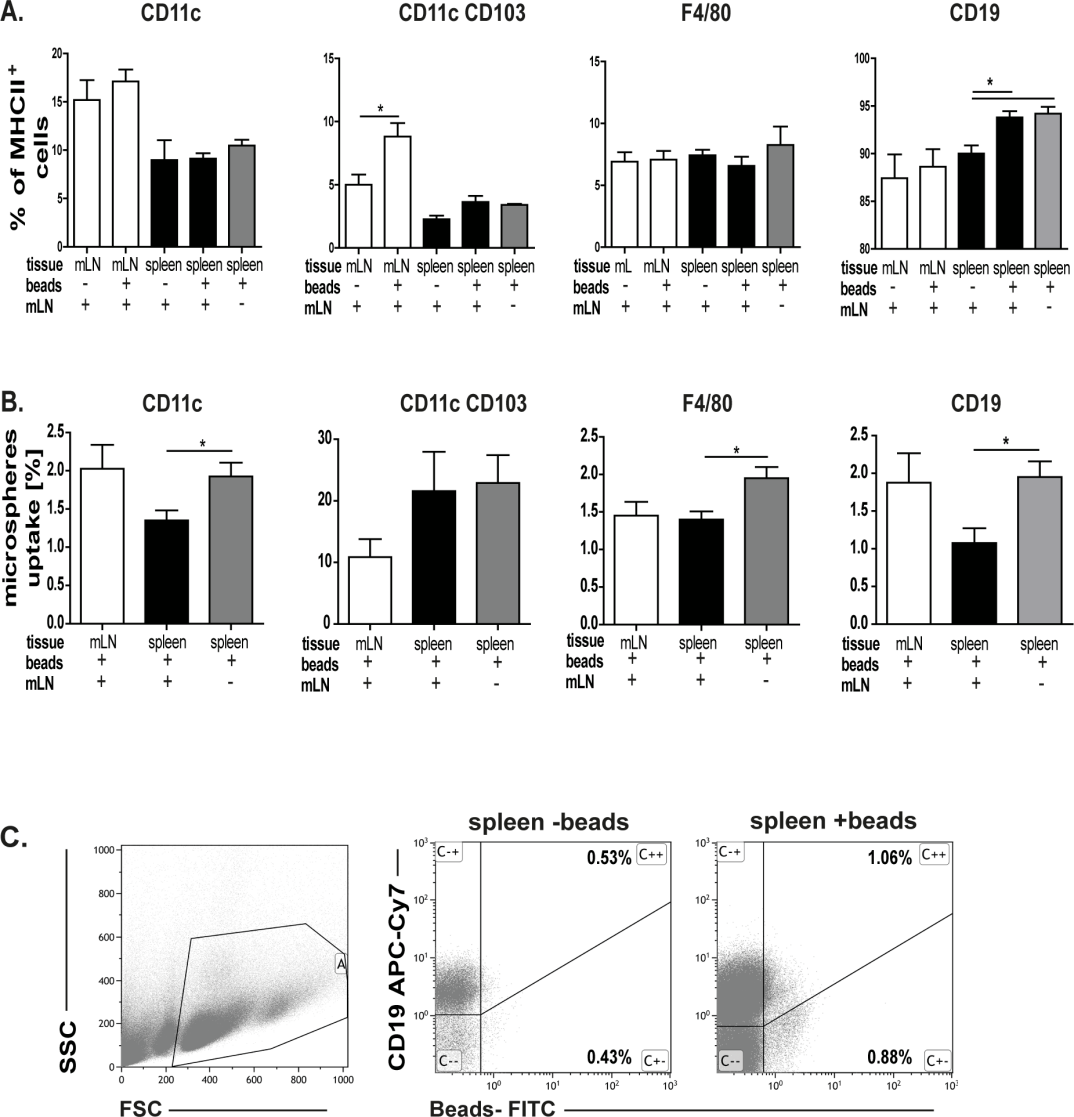
Microspheres uptake. **A.** 10^12^ fluorescence labelled microspheres were orally administered and after 4h cell subsets in the mLN and the spleen of control and mLN resected mice were analysed. Means and standard error are given from 3–4 independent experiments (significant differences in the paired t-test are indicated by *: P < 0.05). **B.** Uptake of microspheres in mLN and the spleen in sham-operated or mLN resected mice. Three to four independent experiments were performed. Significant differences in the paired t-test are indicated by *P< 0.05.**C.** Gating strategy of bead carrying CD19+ cells showed for the spleen.

In our previous study we demonstrated that orally applied CT induces an immune response in mLN [22]. Furthermore, we also showed that the oral application of CT induces formation of the germinal centres in the spleen. Their formation was even more pronounced when mLN were removed [22]. Thus, we analysed whether splenic B cells encounter orally administered Ags. After oral administration of microspheres the number of antigen presenting cells (APC, Fig 1A) solely or carrying microspheres (Fig 1B and 1C) was determined in the mLN and spleen. In the mLN only the number of CD103+ dendritic cells was increased, whereas the amount of B cells and macrophages did not change when microspheres were applied (Fig 1A). Furthermore, number of splenic B cells increased after microspheres treatment and this expansion was independent of the mLN presence (Fig 1A). Thus, these results indicate that the spleen is not only involved in the immune response to systemic Ags, but also to Ags given orally. Interestingly, in the absence of the mLN, microspheres were found in splenic immune cells to a higher extent (Fig 1B). This suggests that the spleen receives more particular Ags from the gut when the mLN, as a filter station, is absent. In addition, these data indicate that splenic B cells collect Ags from the gut.

### CT/OVA oral challenge increases the memory B cell population in the spleen especially in the absence of the mLN

**Fig 2:**
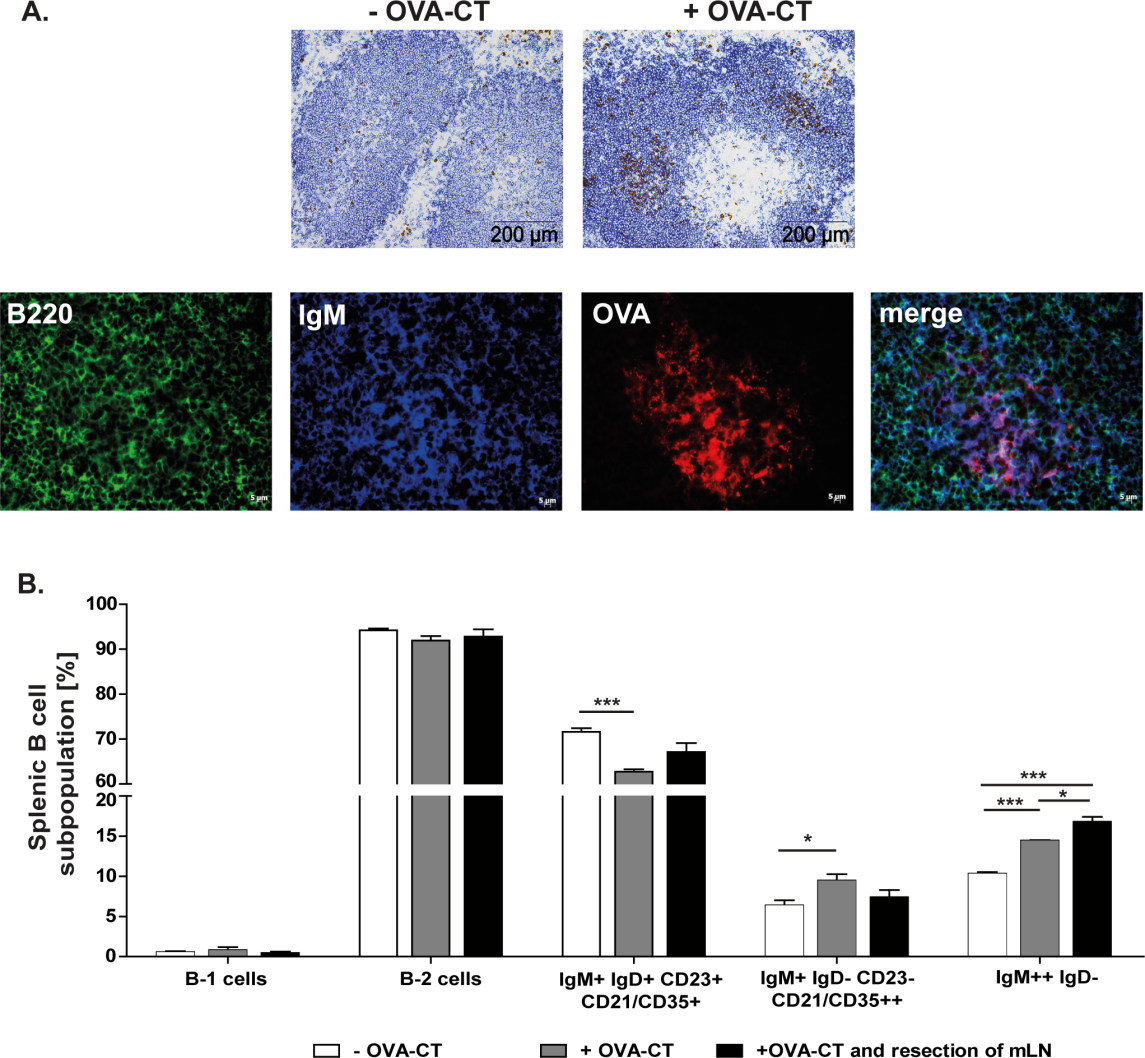
Oral application of OVA-CT induces the proliferation of splenic memory IgM+ and marginal zone B cells. Control and mLN resected mice (n = 3–4) were orally challenged with OVA-CT. 25 days after the challenge the spleen was removed and analysed. **A.** Representative immunohistological staining of cryosections showing incorporated BrdU (brown) and B cells (blue) in the spleen of control mice +/- OVA-CT administration. Focusing on germinal centers after OVA-CT challenge, antigen specific B cells were stained using Alexa Fluor 555 conjugated OVA (red), IgM (blue) and B220 (green). **B.** B1 and B2 cells were distinguished by their IgM, CD19, CD5 and CD11b expression. In addition, B2 cells were separated into follicular B cells (IgM+ IgD+ CD21/35+ CD23+), marginal zone B cells (IgM+ IgD- CD21/35++ CD23-) and memory B cells and marginal zone B cells (IgM++ IgD-). Significant differences in the unpaired t-test are indicated by *P< 0.05; ***P< 0.001.

To determine which B cell population is responsible for the splenic immune response to orally administered Ags mice were challenged with OVA/CT. Stained cryosections showed an increased number of proliferating (BrdU+), antigen specific B cells in germinal centres, indicating an active participation in the immune response against orally applied antigens (Fig 2A). Most of these OVA specific B cells were IgM+ (Fig 2A). In addition, OVA/CT challenge increased the number of antigen experienced B cells in particular IgM++ B cells, which include IgM+ memory B cells and marginal zone B cells. Moreover, removal of the mLN induced even higher proliferation of IgM++IgD- B cells (Fig 2B). Hence, indicating that the oral administration of OVA/CT stimulates proliferation of splenic B cells. And additionally, suggesting that these cells are directly involved in the induction of the splenic immune response to oral Ags.

### Splenic IgM+ B cells respond to oral antigens

**Fig 3:**
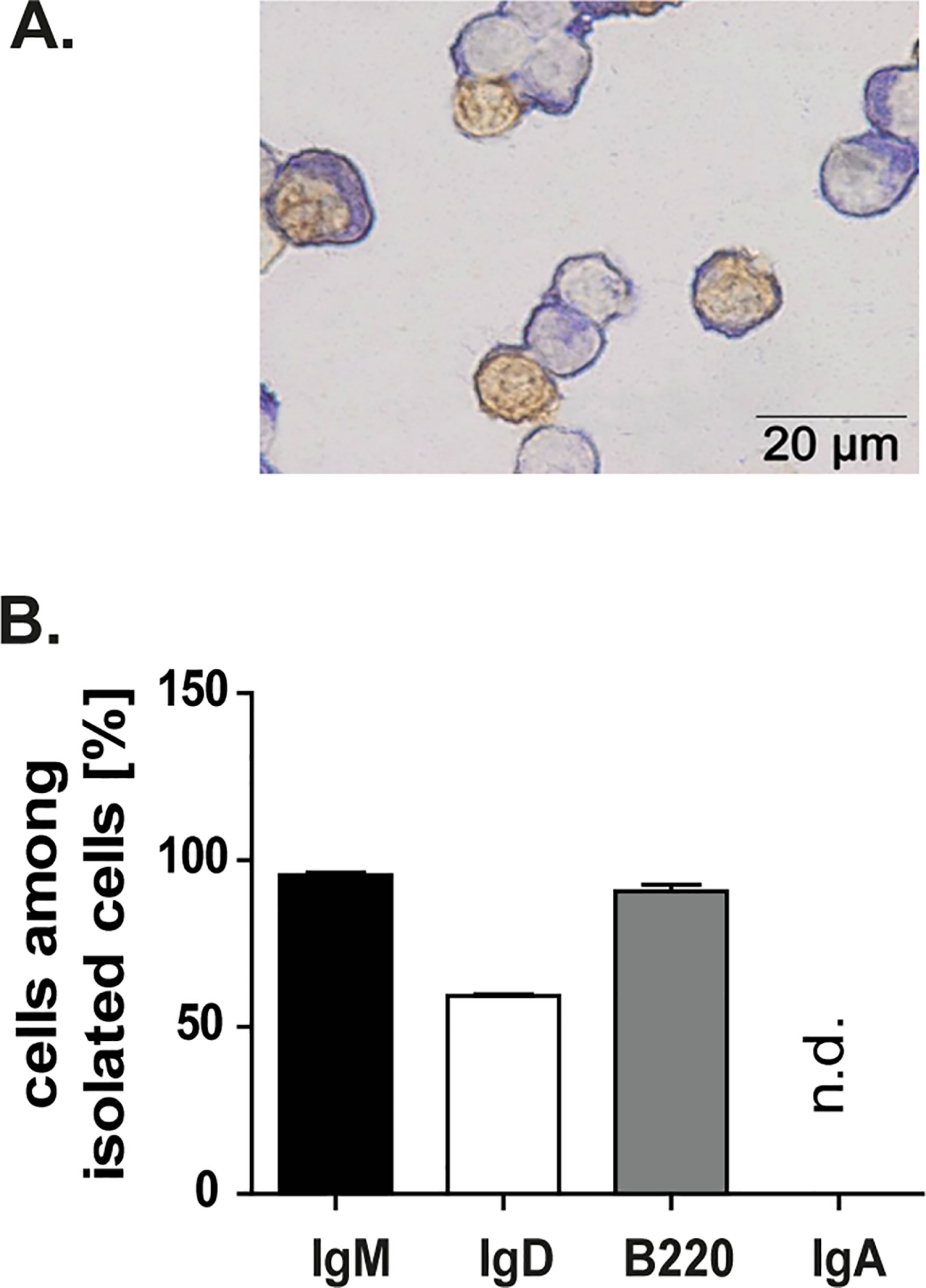
Splenic IgM+ B cells proliferate after orally applied antigen challenge. **A.** Splenic IgM+ B cells of C57BL/6-Ly5.1 mice were isolated using the MACS technique and cytospins of isolated cells were performed. The illustration shows the IgM+ B cells in blue and the proliferating IgM+ B cells (BrdU+ cells) in brown. The purity of the isolated cells was near 100%. **B.** Flow cytometry analysis of isolated cells. Majority of isolated cells were IgM+ B cells. IgA+ B cells were not detected (n.d). Means and standard error of the mean are given for seven independent experiments.

As previously shown, splenic IgM+ B cells responded to oral Ags. Thus, after OVA/CT challenge splenic IgM+ B cells were isolated and analysed. Majority of these cells expressed B220, which can be found on naïve as well as on mature B cells. From analysed IgM+ B cells about 2/3 was IgD+. Conversely, no IgA+ B cells were found in the spleen (Fig 3A and 3B). Because of a cross reaction to the previously used cell sorting antibody, 100% IgG+ B cells were measured. Therefore, IgG+ B cells were not included in the manuscript. Immunohistological staining of incorporated BrdU (Fig 3A) showed that numerous of isolated IgM+ B cells proliferated after encountering OVA/CT. Altogether, these data strongly suggest that the splenic IgM+ B cells play an important role in the response to orally applied antigens.

### Injected splenic IgM+ B cells are mainly found in the gut

**Fig 4:**
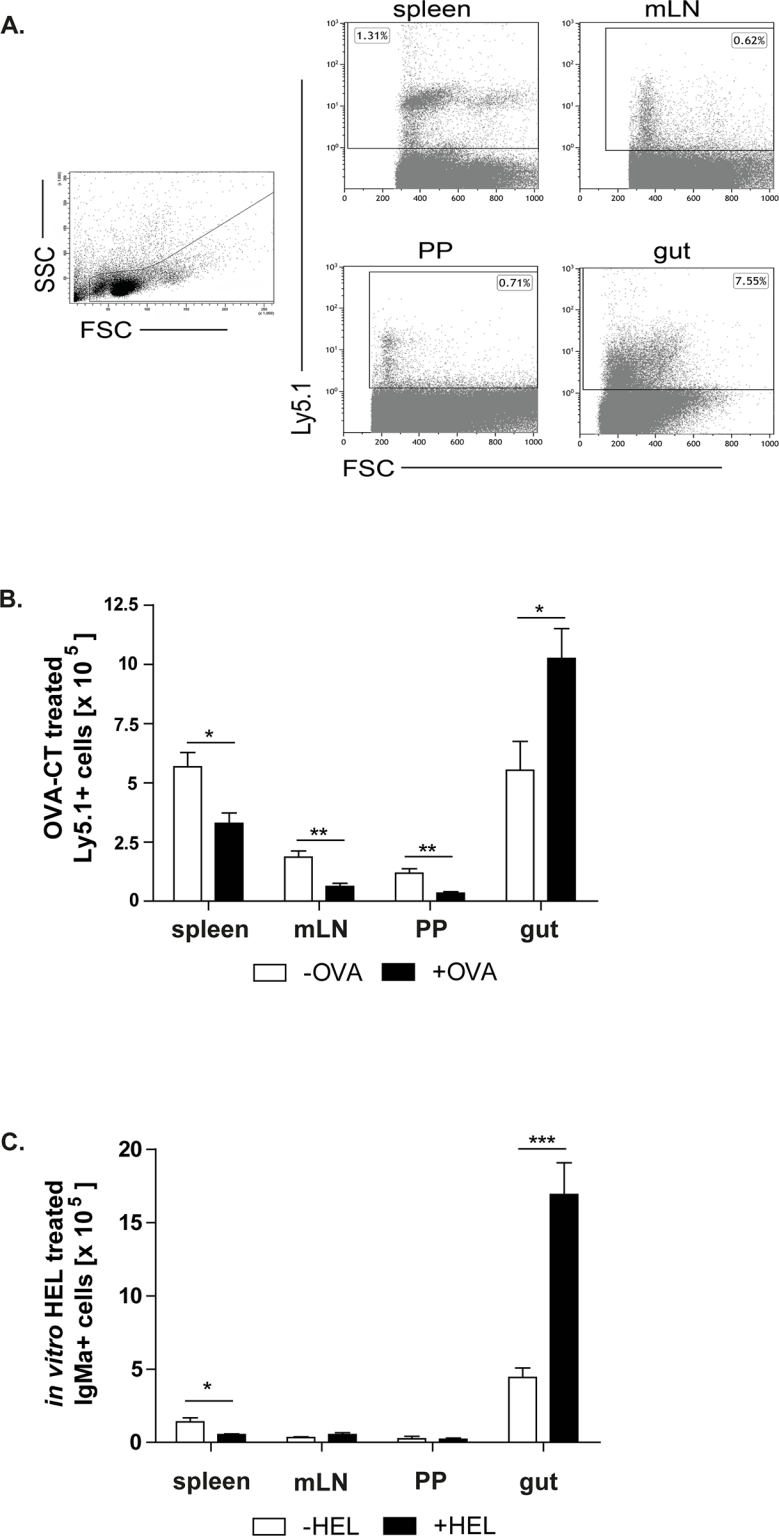
Splenic IgM+ B cells migrate into different lymphoid tissues. Isolated OVA-CT treated IgM+ C57BL/6-Ly.5.1 cells were injected into non-treated (-OVA) and treated (+OVA) mice. Migration of these cells into the gut, PPs, mLN and spleen was analysed by flow cytometry. **A.** Gating strategy of C57BL/6-Ly5.1 injected cells showed for the spleen, mLN, PPs and gut. **B.** Inoculated IgM+ B cells migrated into all analysed tissues. Means and standard error are given from 4–5 independent experiments (significant differences in the unpaired t-test are indicated by *: P < 0.05; **: P < 0.01). **C.** The number of HEL specific B cells was analysed after the cell transfer into WT recipients. Splenic IgM+ B cells were isolated from the MD4 mice, *in vitro* HEL stimulated and injected into non-treated (-HEL) and treated (+HEL) mice. Means and standard error are given from 4–8 independent experiments (significant differences in the unpaired t-test are indicated by *: P < 0.05; ***: P < 0.001).

Splenic IgM+ B cells were isolated from untreated or OVA/CT treated C57BL6/Ly5.1 mice and intravenously injected into OVA treated or non-treated WT recipients. To determine the location where injected cells migrated, various lymphoid tissues were analysed 4 days after the cell transfer (Fig 4, S2 Fig). Low numbers of untreated IgM+ B cells were already found in the analysed tissues (S2A Fig). Furthermore, OVA treatment of the recipient mice did not increase the migration rate of the injected untreated IgM+ B cells (S2 Fig). After the transfer of OVA treated IgM+ B cells more of these cells entered mLN and PPs compared to the migration rate of untreated IgM+ B cells in the same organs (Fig 4B, S2 Fig). However, the spleen and the gut were sites where injected cells mostly migrated. The highest number of injected B cells was found in the gut and their number was even more pronounced when recipients were challenged with OVA (Fig 4B). These data suggest that splenic IgM+ B cells preferentially migrate into the sites where Ags enter the system. To exclude the possibility that the isolated splenic IgM+ B cells were Ag primed and migrated from the gut back to the spleen, *in vitro* stimulated or unstimulated IgM+ B cells isolated from MD4 mice were additionally utilized (Fig 4C, S2C Fig). These splenic B cells carry a hen egg lysozyme (HEL) specific B cell antigen receptor. Splenic IgM+ B cells were isolated, +/- HEL stimulated and subsequently injected into WT recipients to analyse their migration pattern. As expected, these cells migrated into the spleen, PPs and mLN. However, most of these cells were again found in the gut (Fig 4C, S2C Fig).

### Splenic IgM+ B cells mainly differentiate into IgA+ plasma cells in the gut

As described above, we have shown that injected splenic IgM+ B cells migrated into the spleen and the gut. Therefore, we analysed whether these cells underwent the class switch and which particular Ig isotype was expressed on their surface.

**Fig 5:**
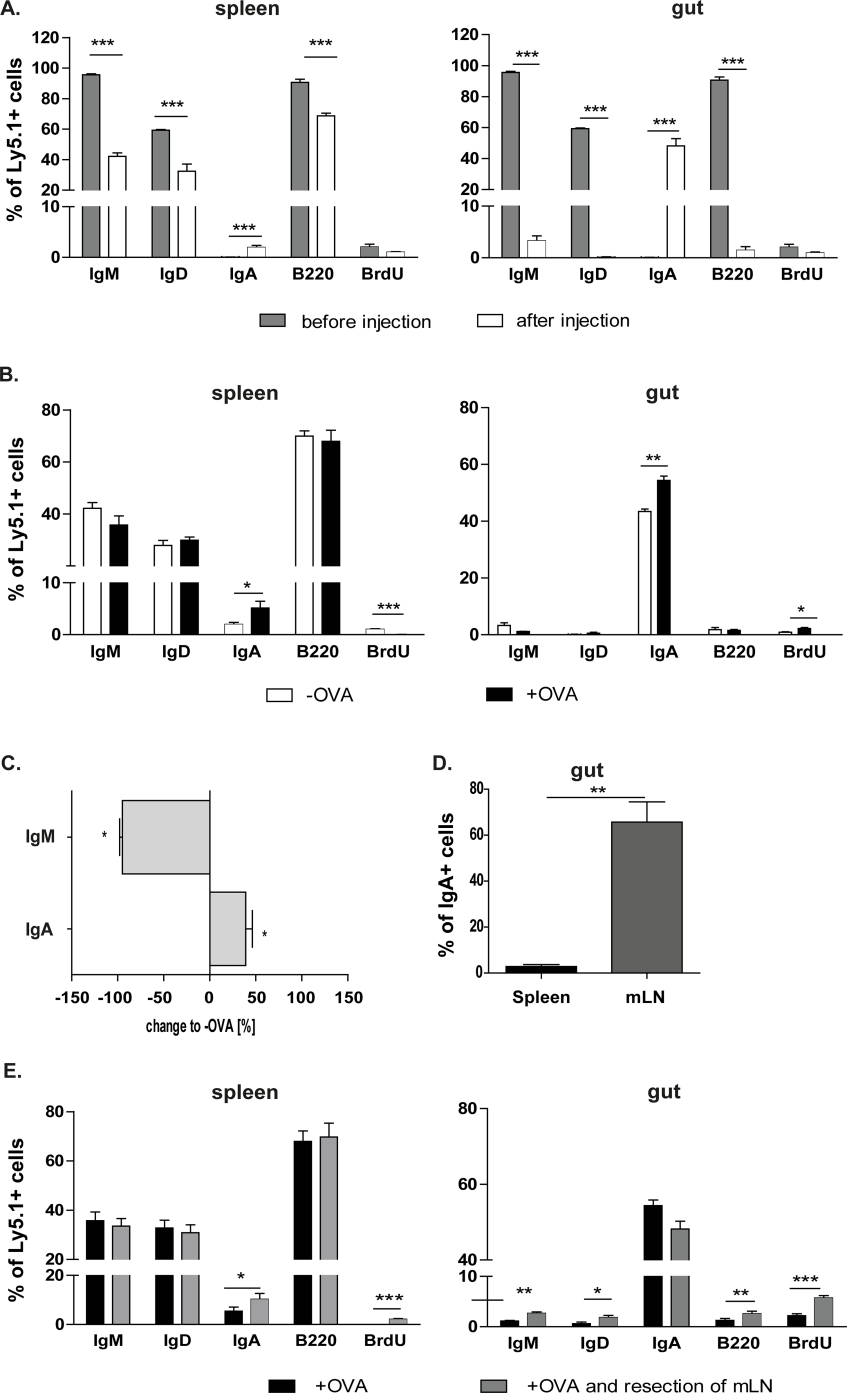
IgM+ B cells that migrate into the gut are OVA-specific IgA+ plasma cells. The mLN resection increases IgA expression in the spleen, but not in the gut. Isolated OVA-CT treated IgM+ C57BL/6-Ly.5.1 cells were analysed for their immunoglobulin expression by flow cytometry. **A.** Comparison of isolated cells before and after the transfer into recipients. After migration of the injected cells into the spleen and the gut percentages were compared to the initial cell population. **B.** IgM+ B cells were injected into non-treated (-OVA) and treated (+OVA) mice and the percentage of different markers expressed on these cells after migrating in the spleen and the gut were analysed using flow cytometry. Significant differences in the unpaired t-test are indicated by *P< 0.05; **P< 0.01; ***P< 0.001. **C.** The serum of non-treated (-OVA) and treated (+OVA) animals, which received splenic IgM+ B cells, was tested in ELISA for OVA-specific antibodies. OVA-specific antibodies of non-treated animals are equalized as 100%. The measured OVA-specific antibodies of treated animals are calculated as a % change to non-treated (-OVA) mice (x-axis). Means and standard error are given from 3–4 independent experiments (significant differences in the unpaired t-test are indicated by *: P < 0.05). **D.** IgM+ B cells of OVA/CT treated mice were isolated from the spleen or the mLN and co-transferred to WT mice. After OVA treatment intestines of these mice were analysed and IgA+ B cells from the mLN and spleen were measured. Two independent experiments were performed (n = 4). Significant differences in the paired t-test are indicated by **P< 0.01. **E.** Recipients underwent the mLN resection. After splenic IgM+ B cell transfer recipients were orally challenged with OVA. Four independent experiments were performed. Significant differences in the unpaired t-test are indicated by *P< 0.05; **P< 0.01; ***P< 0.001.

Inoculated splenic IgM+ B cells, which migrated into the spleen, displayed downregulated expression of IgM, IgD and B220. In addition, these B cells showed upregulated IgA expression (Fig 5A, S2 Fig). However, upregulated IgA expression was more pronounced in B cells that migrated into the intestine. Injected OVA/CT stimulated IgM+ B cells expressed only IgA in the gut, whereas no other markers were detected (Fig 5B). Therefore, these findings indicate that injected IgM+ B cells are able to differentiate into plasma cells and produce IgA. OVA administration boosted the IgA class switch in both the spleen and the gut (Fig 5A and 5B). This was observed in the serum of the cell transfer recipients as well. Namely, orally administered OVA led to a vast decline of OVA-specific IgM, while OVA-specific IgA increased (Fig 5C). To determine the contribution of splenic IgA cells in the gut, co-transfer experiments of OVA/CT treated IgM+ B cells isolated from the spleen or the mLN were performed. As expected, IgA+ B cells originally isolated from the mLN were detected in much higher percentages in the gut compared to IgA+ B cells isolated from the spleen (Fig 5D).

### The mLN resection leads to more IgM+ B cells in the gut

Furthermore, the role of the mLN on the immunoglobulin class switch of injected IgM+ B cells in the gut was assessed. Therefore, mLN were removed and isolated splenic IgM+ B cells were inoculated and analysed after OVA challenge.

Surprisingly, after the mLN resection more IgA+ B cells were found in the spleen, while the other isotypes were not affected (Fig 5E). In contrast, more IgM+, IgD+ and B220+ B cells were detected in the gut, whereas the class switch to IgA+ B cells was reduced. These findings underline the important function of the mLN in the isotype class switch of splenic IgM+ B cells and in the induction of the gut homing.

### HEL increases the activation of injected splenic IgM+ B cells

**Fig 6:**
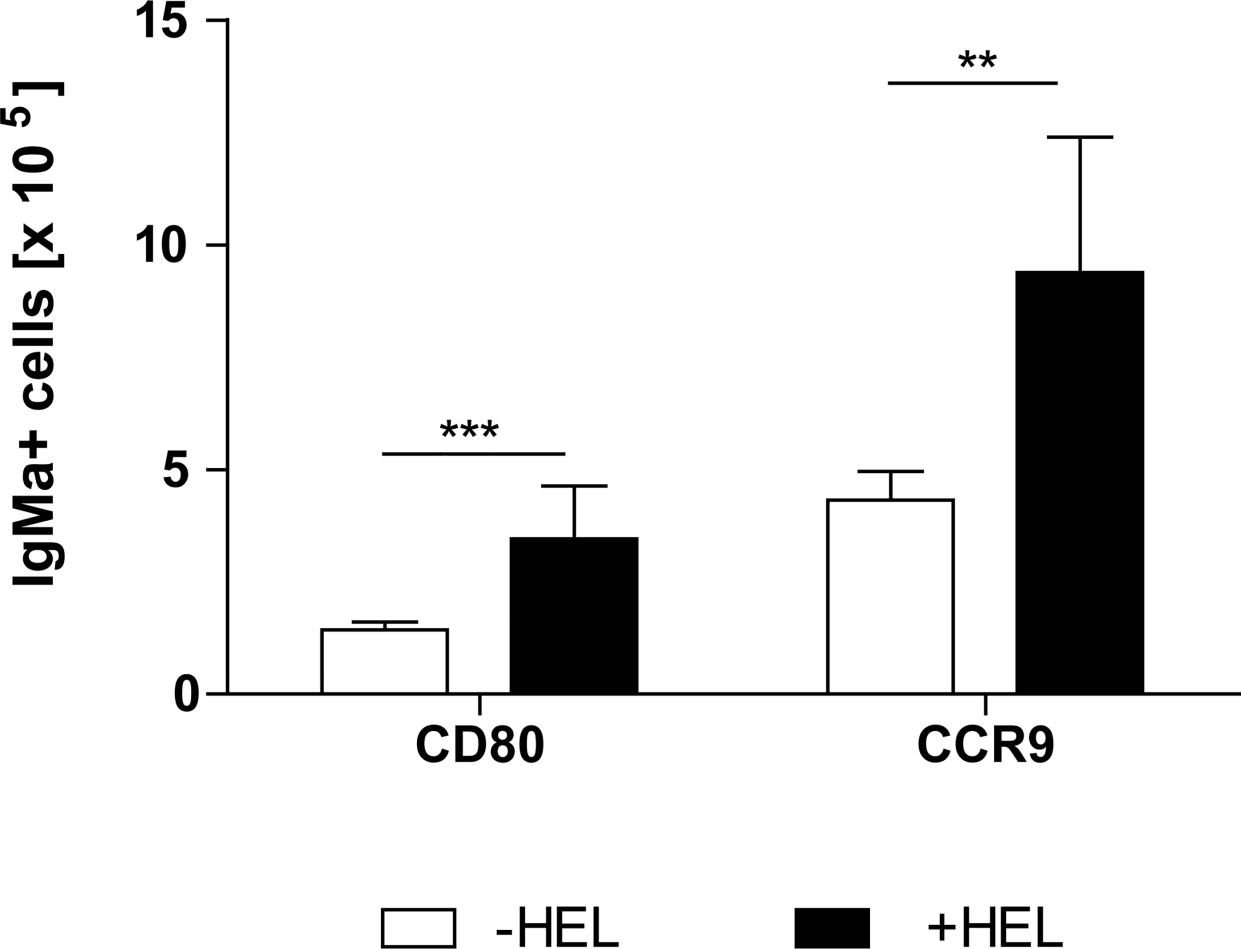
Oral administration of HEL activates transferred IgM+ B cells in the gut. HEL specific IgM+ B cells from MD4 mice were isolated and stimulated *in vitro* for 24h with HEL. Isolated cells were injected into WT mice. WT mice were subsequently challenged with HEL. Expression of CD80 and CCR9 increased after oral HEL treatment in the gut. Significant differences in the unpaired t-test are indicated by **P< 0.01; ***P< 0.001.

As already described above, the *in vitro* HEL stimulated inoculated splenic B cells migrated in the gut (Fig 4C). Thus, we analysed whether these cells were activated and their homing properties altered. Activation and homing properties of these cells were determined by expression analysis of two markers: CD80 and CCR9. CD80 is a marker found on activated B cells, while CCR9 is the gut homing receptor. The expression of the CD80 and CCR9 on previously *in vitro* HEL stimulated splenic IgM+ B cells that migrated into the gut increased in recipient mice that were orally stimulated with HEL (Fig 6). However, when recipients received non HEL treated IgM+ B cells the expression of CD80 and CCR9 on these cells was not changed (S2C Fig). Hence, these results suggest that oral administration of the Ag enhances the activation of these cells in the gut.

## Discussion

Due to the high risk of developing sepsis after splenectomy, the importance of the spleen in the induction of the immune response is widely recognised [38]. However, the role of the spleen in the defence against Ags from the gut has not been studied in detail. Nevertheless, it is known that gastrointestinal disorders such as coeliac disease or inflammatory bowel disease are associated with splenic dysfunction or cell activation [19, 39, 40]. The gut Ags are taken up by intestinal epithelial cells, macrophages or DCs in the intestinal mucosa. Soluble Ags or those collected by DCs are transferred into mLN, where the immune response to these Ags is induced [41–43]. Subsequently, activated cells from mLN migrate back to sites where these Ags were collected [44]. However, to our knowledge, the involvement of other lymphoid organs in this process has not been analysed in detail. In our previous study we have shown that the spleen encounters Ags departed from the gut. In addition, we also observed increased presence of IgA+ B cells in the intestinal mucosa. Therefore, the question arose whether the spleen is the source of these cells [22]. In this study we demonstrated that the spleen is indeed partially involved in the induction of the immune response to Ags sampled in the gut. The role of the spleen was even more pronounced when mLN were removed. Ags that enter the systemic circulation are captured by B cells located in the splenic marginal zone. Thus, our data displayed that Ags collected in the intestinal mucosa were not only restricted to mLN and PPs, but also entered the blood flow and reached the spleen, which then participated in the induction of the immune response to these Ags. This indicated that not only PPs and mLN are involved in the immune response to intestinal antigens, but the spleen as well. Other lymphoid organs, such as peripheral lymph nodes, were not affected (own observation). The biological relevance of this process might be to ensure immunological response to Ags that escaped the gut immune system. The spleen is directly integrated in the blood circulatory system and therefore ideal as a “backup” site to initiate the immune response [9]. Di Sabatino and colleagues showed that even diseases such as rheumatoid arthritis are affected by the hyposplenism [19]. This implicates that the spleen is involved in the development and/ or maintenance of this autoimmune disease and underlines the hypothesis that the spleen has important role in the induction of the immune response [19, 45, 46].

Furthermore, we demonstrated that activated B cells leave spleen and mostly migrate into the site of antigen entrance. To leave the spleen, splenic activated B cells need to express homing molecules, which will guide them to certain sites. In this study, we detected a noticeable population of splenic B cells expressing the CCR9, one of the gut homing receptors. In addition, other groups also demonstrated that splenic cells express α4β7-integrin and CCR9 [47, 48], the most dominant molecules for the gut homing [49, 50]. This indicates that there is not only a direct connection between spleen and gut regarding their Ag supply, but also in their induction of the same homing receptors.

Splenic B cells found in the intestine were mostly IgA+ plasma cells. The current study could not clarify, whether these cells underwent Ig class switch in the gut or elsewhere. However, one possibility could be a class switch in the spleen itself, as increased numbers of IgA secreting cells in the spleen were detected after immunization using various administration routes [27]. However, it is also very likely that splenic IgM+ B cells migrate into the gut and there differentiate into IgA+ plasma cells in the response to the microenvironment. Cytokines such as IL-6, IL-10, IL-4 and TGFß-1 are commonly present in the intestine and it is know that this cytokine milieu favours the IgA class switch [2, 3]. Moreover, the cytokine microenvironment of the spleen and other peripheral organs such as the skin differs from that of the gut [51, 52]. In mLN and PPs, which are closely associated with the gut, the microenvironment is also enriched with TGFß-1 [35]. Thus, the mLN are probably one site where splenic IgM+ B cells might switch into IgA+ B cells and from there migrate into the gut. In addition, after the mLN resection the amount of IgA+ B cells decreased and the number of IgM+ B cells increased in the intestinal lamina propria. Furthermore, in this and our previous study we have shown increased activation of IgM+ B cells after mLN resection [22]. Therefore, the mLN represents an important organ for the control of lymphocyte activation in the periphery. Altogether, our data suggest that splenic IgM+ B cells before migrating in the gut undergo the Ig class switch into IgA+ B cells in the mLN, which also controls their expansion.

Another important factor involved in the isotype class switch and cell migration into the gut is the oral application of the antigen. Our results showed that orally administered Ags enhance the IgA class switch and expansion of IgA+ B cells. This was expected, as promoted immune response at the site of the antigen entrance could prevent systemic dissemination of these antigens. In line with this, there are other reports showing that Ags uptake amplifies the immune response [53]. Therefore, B cell IgA class switch and expansion at the site of antigen uptake may prevent pathogen invasion.

In conclusion, our results suggest that the spleen is playing a role in the gut immune response. This secondary lymphoid organ can supply the intestine with cells, which can mimic the “originally gut derived cells”. Splenic IgM+ B cells differentiate into IgA+ plasma cells and promote the immune response to invading pathogens from the gut lumen. The class switch into IgA+ B cells is supported by the mLN in which these cells are shaped to control the immune response in the intestine.

## Supporting information

S1 Fig Isolation and transfer of splenic IgM+ B cells. **A.** Time scale of the isolation protocol of IgM+ primed B cells after orally applied antigen: C57BL/6-Ly5.1 mice received OVA and CT orally. To evaluate the proliferation of the isolated cells, bromodesoxyuridine (BrdU) was applied intravenously and in the drinking water. On day 25, IgM+ B cells were isolated via MACS. **B**. mLN intact and resected groups received 15 x 10^6^ isolated IgM+ primed B cells. Control groups were left untreated (-OVA), whereas other groups orally received OVA on day 1 (+OVA). Four days after the inoculation mice were sacrificed and various tissues were analysed.
(TIF)

S2 Fig Migration pattern of splenic IgM+ B cells without OVA-CT treatment. Isolated non OVA-CT treated IgM+ C57BL/6-Ly.5.1 cells were injected into non-treated (-OVA) and treated (+OVA) mice and the number of these cells in various tissues was analysed by flow cytometry. **A.** Analysis of cell migration in the spleen, mLN, PP and the gut. **B.** The percentage of different markers and immunoglobulines expressed on transferred cells in the spleen and gut analysed by flow cytometry. Means and standard error are given from 3 independent experiments. **C.** The number of HEL specific IgM+ B cells in the spleen, mLN, PP and the gut was analysed after the cell transfer into WT recipients without HEL stimulation. Expression of CD80 and CCR9 was not altered after oral HEL treatment in the gut. Means and standard error are given from 3–6 independent experiments.
(TIF)

## Abbreviations

Ags: Antigens
CT: cholera toxin
mLN: mesenteric lymph nodes
