# Supplementary figures and images for: Participation of the spleen in the IgA immune response in the gut
Source: PLoS One. 2018 Oct 4;13(10):e0205247. doi: 10.1371/journal.pone.0205247 (PMC6171922; doi:10.1371/journal.pone.0205247)

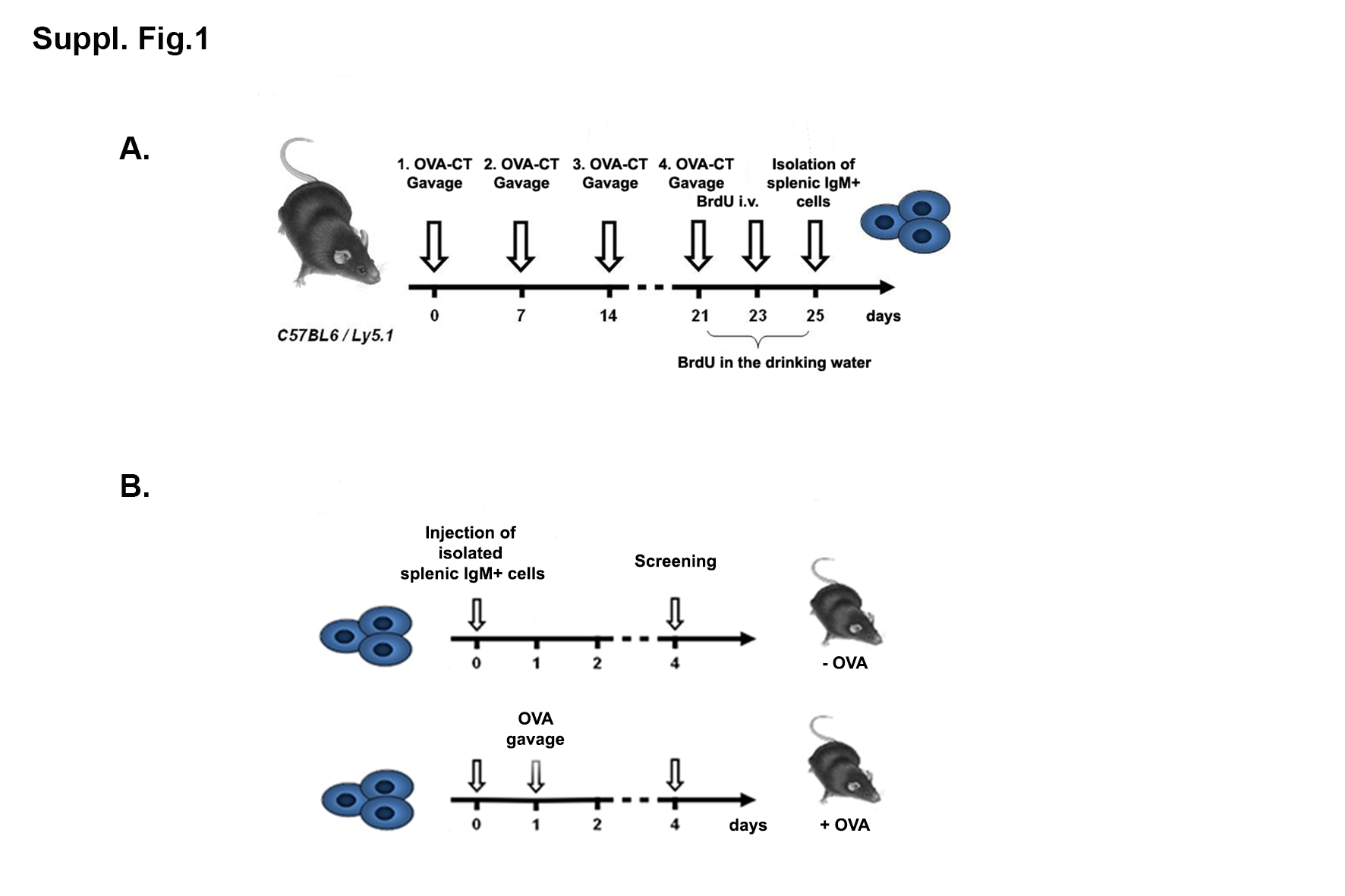

Supplement: S1 Fig — A. Time scale of the isolation protocol of IgM+ primed B cells after orally applied antigen: C57BL/6-Ly5.1 mice received OVA and CT orally. To evaluate the proliferation of the isolated cells, bromodesoxyuridine (BrdU) was applied intravenously and in the drinking water. On day 25, IgM+ B cells were isolated via MACS. B. mLN intact and resected groups received 15 x 106 isolated IgM+ primed B cells. Control groups were left untreated (-OVA), whereas other groups orally received OVA on day 1 (+OVA). Four days after the inoculation mice were sacrificed and various tissues were analysed. (TIF) [file pone.0205247.s001.tif]

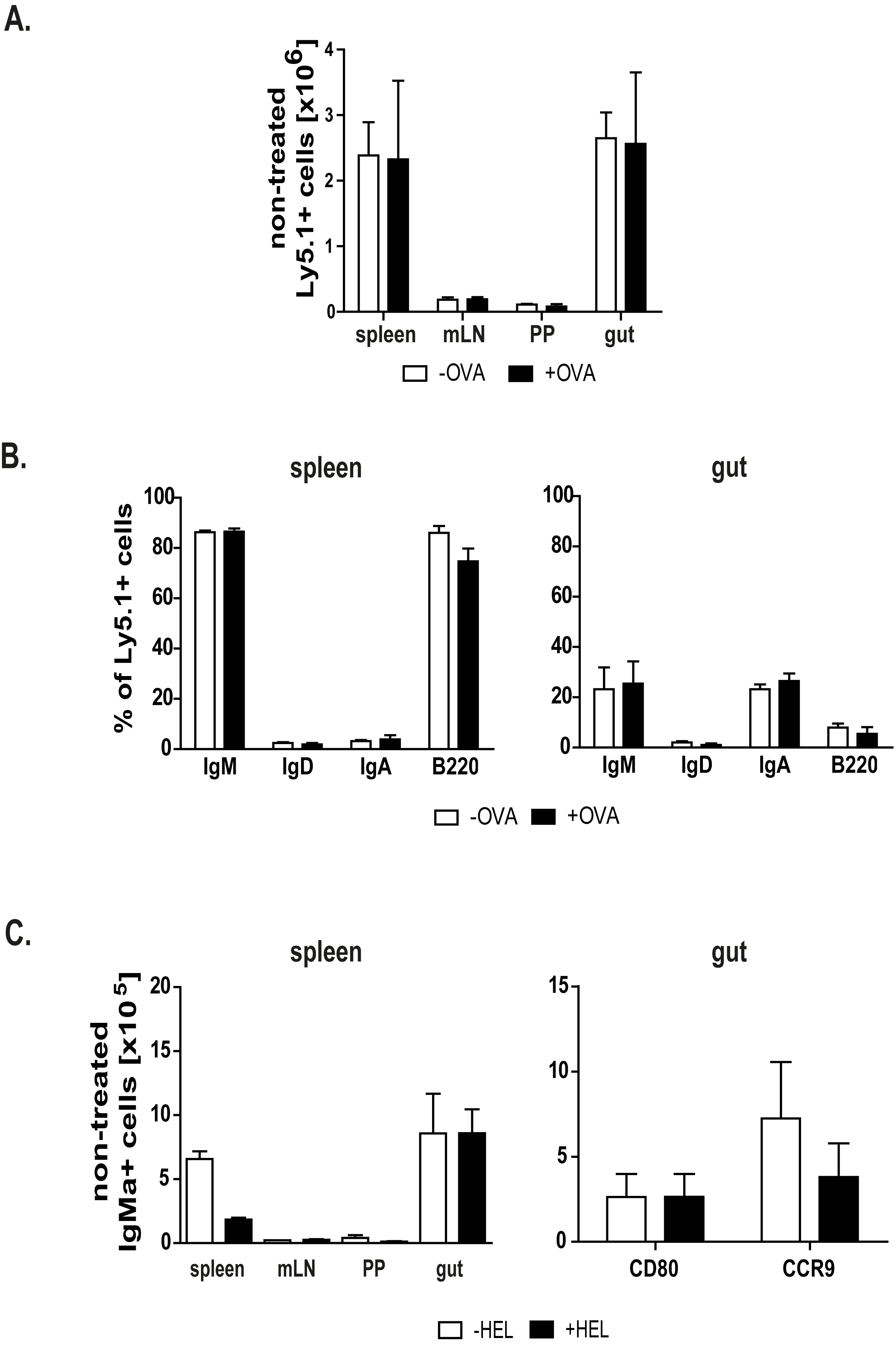

Supplement: S2 Fig — Isolated non OVA-CT treated IgM+ C57BL/6-Ly.5.1 cells were injected into non-treated (-OVA) and treated (+OVA) mice and the number of these cells in various tissues was analysed by flow cytometry. A. Analysis of cell migration in the spleen, mLN, PP and the gut. B. The percentage of different markers and immunoglobulines expressed on transferred cells in the spleen and gut analysed by flow cytometry. Means and standard error are given from 3 independent experiments. C. The number of HEL specific IgM+ B cells in the spleen, mLN, PP and the gut was analysed after the cell transfer into WT recipients without HEL stimulation. Expression of CD80 and CCR9 was not altered after oral HEL treatment in the gut. Means and standard error are given from 3–6 independent experiments. (TIF) [file pone.0205247.s002.tif]
